# New Onset Diabetes Mellitus in Living Donor versus Deceased Donor Liver Transplant Recipients: Analysis of the UNOS/OPTN Database

**DOI:** 10.1155/2013/269096

**Published:** 2013-09-24

**Authors:** Anitha D. Yadav, Yu-Hui Chang, Bashar A. Aqel, Thomas J. Byrne, Harini A. Chakkera, David D. Douglas, David C. Mulligan, Jorge Rakela, Hugo E. Vargas, Elizabeth J. Carey

**Affiliations:** ^1^Division of Hepatology, Mayo Clinic Hospital, 5777 E. Mayo Boulevard, Phoenix, AZ 85054, USA; ^2^Division of Health Sciences Research, Mayo Clinic Hospital, 5777 E. Mayo Boulevard, Phoenix, AZ 85054, USA; ^3^Division of Nephrology, Mayo Clinic Hospital, 5777 E. Mayo Boulevard, Phoenix, AZ 85054, USA; ^4^Division of Transplant Surgery, Mayo Clinic Hospital, 5777 E. Mayo Boulevard, Phoenix, AZ 85054, USA

## Abstract

New onset diabetes after transplantation (NODAT) occurs less frequently in living donor liver transplant (LDLT) recipients than in deceased donor liver transplant (DDLT) recipients. The aim of this study was to compare the incidence and predictive factors for NODAT in LDLT versus DDLT recipients. The Organ Procurement and Transplant Network/United Network for Organ Sharing database was reviewed from 2004 to 2010, and 902 LDLT and 19,582 DDLT nondiabetic recipients were included. The overall incidence of NODAT was 12.2% at 1 year after liver transplantation. At 1, 3, and 5 years after transplant, the incidence of NODAT in LDLT recipients was 7.4, 2.1, and 2.6%, respectively, compared to 12.5, 3.4, and 1.9%, respectively, in DDLT recipients. LDLT recipients have a lower risk of NODAT compared to DDLT recipients (hazard ratio = 0.63 (0.52–0.75), *P* < 0.001). Predictors for NODAT in LDLT recipients were hepatitis C (HCV) and treated acute cellular rejection (ACR). Risk factors in DDLT recipients were recipient male gender, recipient age, body mass index, donor age, donor diabetes, HCV, and treated ACR. LDLT recipients have a lower incidence and fewer risk factors for NODAT compared to DDLT recipients. Early identification of risk factors will assist timely clinical interventions to prevent NODAT complications.

## 1. Introduction

New onset diabetes mellitus after transplantation (NODAT) is a serious metabolic complication with a reported incidence of 10% to 36% in liver transplant recipients [1–8]. The variation in the incidence of NODAT is due to differences in the diagnostic criteria for NODAT, patient characteristics,duration of the study period, and variation of immunosuppressive regimens used. Studies suggest that NODAT in liver transplant recipients is associated with a significant increase in cardiovascular disease, infection, and decreased graft survival [6–10]. Multiple risk factors are known to be associated with NODAT [1–6, 11, 12]. Age, male gender, body mass index (BMI), hepatitis C virus infection (HCV), impaired fasting glucose, immunosuppressive medications, and acute cellular rejection (ACR) episodes are documented as predictive factors for NODAT. The existing literature focuses on the prevalence and risk factors for NODAT in deceased donor liver transplant (DDLT) recipients [1–6]. However, the incidence and predictors of NODAT in living donor liver transplant (LDLT) recipients are not well established. In a single center retrospective study of 84 Chinese LDLT recipients, the incidence of NODAT was 14.9% [13]. The only risk factor identified was body mass index.

Previous studies demonstrated a lower risk of NODAT in LDLT recipients [1, 2]. Suggested contributory factors for this decreased risk include favorable recipient and donor characteristics (younger donor age and lower MELD score and BMI among recipients). There are no confirmatory studies evaluating the lower incidence and predictive factors of NODAT in LDLT recipients. Hence, this study aimed to identify and compare the incidence and risk factors for NODAT in LDLT versus DDLT recipients using a large national database. The incidence of NODAT over a five-year follow-up after liver transplantation was also assessed.

## 2. Methods

The Organ Procurement and Transplant Network/United Network for Organ Sharing (OPTN/UNOS) database from 2004 to 2010 was analyzed. A total of 20,484 recipients with pretransplant diabetes, multiorgan transplant, and retransplantation were excluded. The study group included 902 LDLT and 19,582 DDLT recipients, none of whom had diabetes mellitus prior to liver transplantation. All patients had at least one follow-up recorded in the database. The incidence of NODAT at 6 months and consecutive 5 years after-transplant in both LDLT and DDLT groups was evaluated.

Recipient risk factors included age at transplant, gender, race (African-American (AA), Caucasian, and Hispanic), BMI, etiology of liver disease (alcoholic liver disease (ALD), HCV, nonalcoholic steatohepatitis (NASH), and others), history of diabetes, and model for end-stage liver disease (MELD) score at the time of transplant. Donor risk factors included age, gender, race, and history of diabetes. Transplant related variables included cold ischemia time, treated ACR episodes, steroid induction, and use of calcineurin inhibitor. 

## 3. Definition of NODAT

NODAT was identified in recipients who had at least one record of diabetes during the posttransplant follow-up period. The UNOS Transplant Recipient Follow-up form does not define the precise diagnostic criteria for NODAT. The onset of diabetes is documented as “yes” or “no” on the UNOS follow-up record. 

## 4. Statistical Analysis

Statistical analysis was performed using two-sample *t*-tests to compare continuous variables and chi-square tests to compare categorical variables. The Kaplan-Meier product limit method was used to estimate the survival rate, and log-rank test was used to compare the overall survival between subgroups for each of the potential risk factors. Since the outcome of interest was time to the onset of NODAT after transplant, patients were considered censored in cases of death, graft loss, or loss to follow-up. The maximum follow-up duration was 5 years after transplant. To evaluate the risk factors for NODAT, Cox proportional hazard modeling was employed and the hazard ratios (HR) were reported. Univariate analysis was performed first, and variables with statistical significance were evaluated by multivariate analysis. All *P* values were two sided, and a *P* value of less than or equal to 0.05 was considered statistically significant. All analysis was performed using SAS 9.1 (SAS institute, Cary, NC) and graphs were created using R 2.12.1 (R foundation for statistical computing, Vienna, Austria).

## 5. Results 

A total of 902 LDLT and 19,582 DDLT recipients were included in our study. The baseline demographic statistics for LDLT and DDLT recipients are shown in Table 1. The mean age in the LDLT cohort was 50.2 ± 12.2 and for DDLT was 52.7 ± 10.2 years (*P* < 0.001).

**Figure 1:**
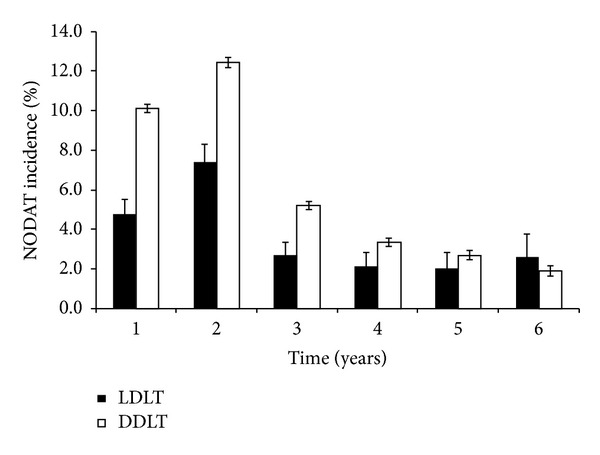
Incidence of NODAT in LDLT and DDLT recipients over time.

The overall incidence of NODAT was 12.2% at 1 year after transplantation. At 1, 3, and 5 years after transplant, the incidence of NODAT in LDLT was 7.4, 2.1, and 2.6%, respectively, compared to 12.5, 3.4, and 1.9%, respectively, in DDLT (Figure 1). The incidence of NODAT decreased as the duration of follow-up increased.

**Figure 2:**
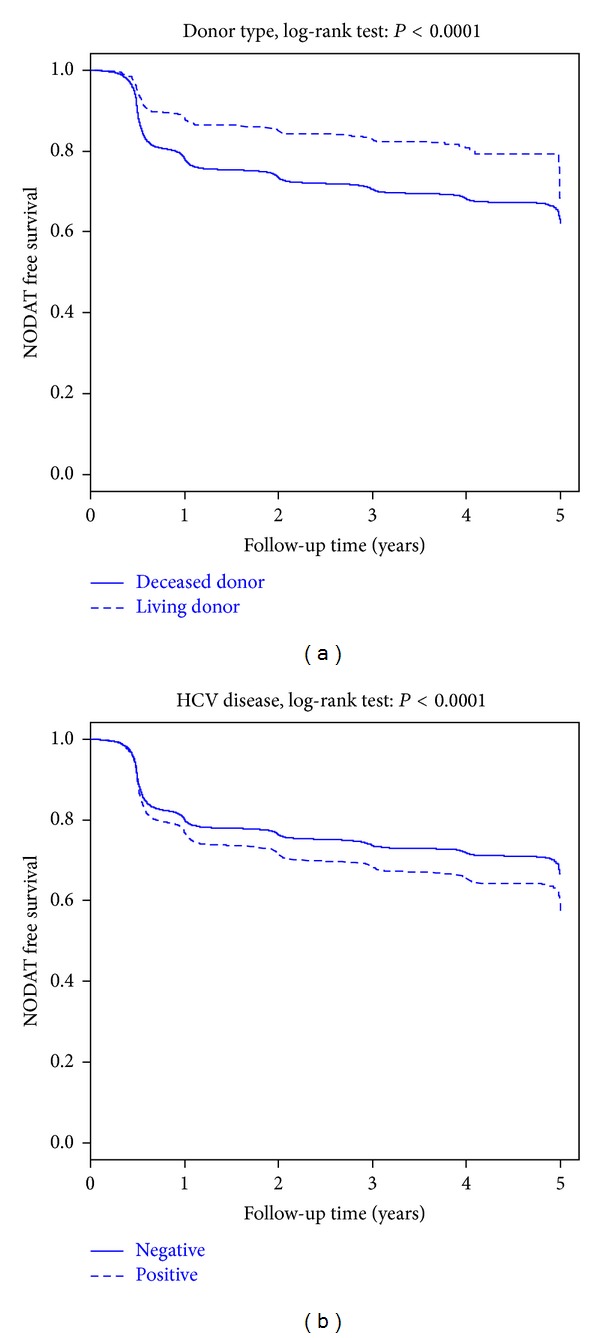
Kaplan-Meier NODAT-free survival curves for LDLT and DDLT recipients according to donor type and HCV.

Kaplan-Meier plots for NODAT-free survival for donor type and HCV liver disease are shown in Figure 2. Higher NODAT-free survival was observed in LDLT compared to DDLT recipients (87.7%, 83%, and 68.3% versus 77.9%, 70.5%, and 62% at 1, 3, and 5 years, resp., (*P* < 0.001)).

Univariate analyses demonstrated recipient age (>50 years versus ≤50 years), recipient race (AA versus others and Hispanic versus others), recipient male gender, recipient BMI (>30 kg/m^2^ versus ≤25 kg/m^2^), donor age (≥60 years versus <60 years), etiology of liver disease (HCV versus others and ALD versus others), and treated ACR episodes as significant risk factors for NODAT in the DDLT cohort. However, subgroup analyses of LDLT recipients demonstrated only ACR (treated ACR versus no ACR) and etiology of liver disease (HCV liver disease versus others and ALD versus others) as significant predictors for NODAT.

Significant risk factors in univariate analyses were included in the multivariate analysis.  Table 2 shows unadjusted and adjusted hazard ratios for developing NODAT among all LT recipients. LDLT recipients had a significantly lower risk of NODAT compared to DDLT recipients (LDLT versus DDLT, HR = 0.63 (0.52–0.75) *P* < 0.001). 

Discrete risk factors for NODAT in LDLT and DDLT recipients were also analyzed separately. The independent risk factors for NODAT in LDLT and DDLT cohorts have been reported in Tables 3 and 4, respectively.

## 6. Discussion

Based on the large multicenter UNOS/OPTN database, the incidence of NODAT at one year after liver transplantation is 7.4% and 12.5% among LDLT and DDLT recipients, respectively, with decreasing incidence of NODAT overtime in both cohorts. Our study identified HCV liver disease and treated ACR events as risk factors for development of NODAT in LDLT recipients. 

We and others have demonstrated a lower risk of NODAT in LDLT recipients [1, 2]. In a previous single center study, we reported a decreased risk of NODAT among LDLT recipients (OR 0.22 (0.05–0.98) *P* = 0.05) [1]. Similarly, Kuo et al.'s review of the UNOS/OPTN database also showed lower risk of NODAT in LDLT recipients (HR = 0.628 (0.512–0.769) *P* < 0.001) [2]. The decreased risk for NODAT among LDLT recipients could be secondary to favorable donor and recipient characteristics as demonstrated in our study. Recipients receiving LDLT compared to DDLT were younger (50.2 versus 52.7, *P* < 0.001) with lower BMI (26.3 kg/m^2^ versus 35.8 kg/m^2^, *P* = 0.0227), lower MELD score (14.6 versus 20.9, *P* < 0.001), and fewer HCV recipients (35.6% versus 50.2%, *P* < 0.001). Also, donors for LDLT recipients were younger compared to deceased donors (37.8 years versus 41.4 years, *P* < 0.001).

We demonstrate HCV as an important risk factor for NODAT in both LDLT and DDLT recipients. Prior studies have reported conflicting results regarding HCV as a predictor of NODAT [2–6, 14–16]. Khalili and colleagues demonstrated HCV as an independent predictor of NODAT in a cohort of 555 DDLT recipients (OR 2.6 (1.2–5.7) *P* = 0.02). A meta-analysis study also revealed increased risk of NODAT associated with HCV liver disease (OR 2.46; 95%CI (1.44, 4.19), [16]. However, the association between HCV and NODAT in LDLT recipients has not been well studied. In a Japanese cohort of 62 HCV and 161 non-HCV LDLT recipients, NODAT occurred more frequently in HCV compared to non-HCV patients (41% versus 22%, *P* = 0.03). However, HCV did not emerge as a significant predictor for NODAT in multivariate analysis [17]. All HCV patients received preemptive antiviral therapy (interferon *α*-2b and ribavirin) starting approximately 1 month after LDLT, and 37 out of 62 (60%) recipients were HCV RNA positive at 6 months after transplant. In contrast, our study showed HCV as an independent risk factor for NODAT (HR 1.18 (2.33–8.15) *P* < 0.001). 

Our investigation also revealed ACR as a significant predictor for NODAT in both LDLT and DDLT recipients. Few studies have demonstrated increased episodes of ACR in DDLT recipients with NODAT [9, 18]. In a single center study of DDLT recipients (*n* = 138), ACR was seen more commonly in the NODAT group compared to controls with no diabetes (50% versus 30%, *P* = 0.03) [9]. However, treatment regimens used for management of ACR episodes were not reported. In another investigation, Navasa et al. observed significantly higher ACR episodes in 102 DDLT recipients with posttransplant diabetes than in nondiabetic recipients (1.5 versus 1.1, *P* < 0.05) at 1 year after transplantation [18]. Our study confirms treated ACR as a predictor for NODAT in both DDLT and LDLT recipients. NODAT-reducing treatment strategies for ACR should be the focus of future research. 

The incidence of NODAT following liver transplantation decreases over time. The long-term incidence of NODAT after liver transplantation in LDLT recipients has not been reported. Navasa and his colleagues also demonstrated a decline in the prevalence of NODAT over time, but the study was limited to a cohort of 88 DDLT recipients followed over a 3-year period [18]. In our study, the decline in the incidence of NODAT may be related to decreased use of maintenance immunosuppression and relatively fewer ACR episodes over time.

Our study has several limitations. First, our analysis may be subjected to reporting error or bias inherent to the large registry database. Second, no standardized diagnostic criteria for identification of NODAT were utilized. Third, there was significant heterogeneity of immunosuppression protocols and antiviral regimens after transplantation across different centers. Fourth, the MELD score reported includes exception points and could be a confounding factor. Lastly, traditional risk factors such as recipient family history of diabetes and pretransplant impaired fasting glucose were not available for analysis.

In summary, the incidence of NODAT at 1 year after liver transplantation for LDLT and DDLT recipients was 7.4% and 12.5%, respectively. LDLT recipients had a lower incidence, fewer risk factors, and higher 5-year NODAT-free survival compared to DDLT recipients. Due to the higher incidence of NODAT in the first six months following liver transplantation, early intervention during this time period may prevent the development of NODAT. Large prospective studies are needed to identify the impact of NODAT on patient and graft outcomes in both LDLT and DDLT recipients.

## Figures and Tables

**Table 1:** Baseline demographic statistics for DDLT and LDLT recipients.

Variables	DDLT (*n* = 19582)	LDLT (*n* = 902)	*P* value
Recipient age, *n* (%)			
≤50	6652 (34)	386 (42.8)	<0.0001
>50	12930 (66)	516 (57.2)	
Recipient gender, *n* (%)			
Female	6350 (32.4)	405 (44.9)	<0.0001
Male	13232 (67.6)	497 (55.1)	
Recipient race, *n* (%)			
Caucasian	14377 (73.4)	761 (84.4)	<0.0001
African American	1784 (9.1)	32 (3.5)	
Hispanic	2320 (11.8)	76 (8.4)	
Others	1101 (5.6)	33 (3.7)	
Recipient BMI, *n* (%)			
≤25	6154 (32.9)	396 (45.7)	<0.0001
25–30	6800 (36.3)	299 (34.5)	
>30	5761 (30.8)	171 (19.7)	
Etiology of liver disease, *n* (%)			
ALD	2481 (12.7)	78 (8.6)	<0.0001
ALF	1070 (5.5)	24 (2.7)	
HCV	9837 (50.2)	324 (35.9)	
NASH	646 (3.3)	17 (1.9)	
Others	5548 (28.3)	59 (50.9)	
MELD score			
Mean (SD)	20.9 (9.8)	14.6 (5.3)	<0.0001
Donor age, *n* (%)			
<40	8754 (44.7)	508 (56.4)	<0.0001
40–60	7926 (39.5)	380 (42.3)	
≥61	3102 (15.8)	13 (1.4)	
Donor gender, *n* (%)			
Female	7876 (40.2)	446 (49.4)	<0.0001
Male	11706 (59.8)	456 (50.6)	
Donor race, *n* (%)			
Caucasian	13304 (67.9)	766 (84.9)	<0.0001
African American	3094 (15.8)	26 (2.9)	
Hispanic	2567 (13.1)	81 (9)	
Others	617 (3.2)	29 (3.2)	
Cold ischemia time, *n* (%)			
<6 h	6385 (32.6)	658 (93.2)	<0.0001
6–12 h	11105 (56.7)	12 (1.7)	
>12 h	839 (4.6)	36 (5.1)	
Episode of rejection, *n* (%)			
Yes (with treatment)	967 (4.9)	33 (3.7)	0.1267
Yes (no treatment)	250 (1.3)	9 (1)	
No	17265 (88.2)	807 (89.4)	
Missing	1100 (5.6)	53 (5.9)	
Steroid induction, *n* (%)			
Yes	10780 (55)	650 (72)	<0.0001
No	7141 (36.5)	227 (25.2)	
Missing	1661 (8.5)	25 (2.8)	
Calcineurin inhibitor use, *n* (%)			
Yes	18806 (96)	881 (97.7)	0.0159
No	776 (4)	21 (2.3)	
Patient survival time (days)			
Mean (SD)	954.5 (608.5)	1082.5 (642.6)	<0.0001

DDLT: deceased donor liver transplant; LDLT: living donor liver transplant; BMI: body mass index; ALD: alcohol liver disease; ALF: acute liver failure; HCV: hepatitis C virus; NASH: nonalcoholic steatohepatitis; MELD: model for end-stage liver disease.

**Table 2:** Association of risk factors for development of NODAT for all LT recipients (*n* = 20, 484).

Variable		Univariate HR (95% CI)	*P* value	Multivariate HR (95% CI)	*P* value
Donor type	Living versus deceased	0.55 (0.46–0.65)	<0.0001	0.63 (0.52–0.75)	<0.0001

Recipient age	>50 versus ≤50	1.23 (1.22–1.38)	<0.0001	1.14 (1.07–1.22)	0.0002

Recipient gender	Male versus female	1.16 (1.10–1.24)	<.0001	1.13 (1.06–1.20)	0.0003

Recipient race	AA versus others	1.91 (1.03–1.38)	0.021	1.07 (0.92–1.26)	0.3833
	Caucasian versus others	1.03 (0.91–1.17)	0.654	0.95 (0.84–1.09)	0.485
	Hispanic versus others	1.17 (1.01–1.35)	0.038	1.05 (0.90–1.22)	0.526

Recipient BMI	>30 versus ≤25	1.36 (1.26–1.46)	<0.0001	1.27 (1.18–1.37)	<0.0001
	25–30 versus ≤25	1.20 (1.12–1.28)	<0.0001	1.15 (1.07–1.23)	0.0002

Donor age	≥60 versus <60	1.26 (1.19–1.35)	<0.0001	1.21 (1.12–1.30)	<0.0001

Etiology of liver disease	ALD versus others	1.33 (1.20–1.46)	<0.0001	1.26 (1.13–1.40)	<0.0001
	HCV versus others	1.46 (1.36–1.57)	<0.0001	1.38 (1.27–1.49)	<0.0001
	NASH + cryptogenic cirrhosis versus others	1.58 (1.42–1.77)	<0.0001	1.47 (1.31–1.65)	<0.0001

Rejection	Treated rejection versus no rejection	3.35 (3.06–3.65)	<0.0001	3.50 (3.2–3.84)	<0.0001

**Table 3:** Independent risk factors for NODAT for all LDLT recipients (*n* = 902).

Variable		Univariate HR (95% CI)	*P* value	Multivariate HR (95% CI)	*P* value
Rejection episodes	Treated rejection versus no rejection	4.12 (2.26–7.50)	<0.0001	4.36 (2.33–8.15)	<0.0001

Etiology of liver disease	ALD versus others	2.44 (1.31–4.55)	0.0049	2.45 (1.29–4.68)	0.0065
	HCV versus others	3.67 (2.46–5.48)	<0.0001	3.43 (2.27–5.17)	<0.0001
	NASH + cryptogenic cirrhosis versus others	1.16 (0.45–2.96)	0.759	1.23 (0.48–3.15)	0.6653

LDLT: living donor liver transplant; ALD: alcohol liver disease; HCV: hepatitis C virus; NASH: nonalcoholic steatohepatitis; HR: hazard ratio; CI: confidence interval.

**Table 4:** Independent risk factors for development of NODAT for all DDLT recipients (*n* = 19, 582).

Variable		Univariate HR (95% CI)	*P* value	Multivariate HR (95% CI)	*P* value
Recipient age	>50 versus ≤50	1.27 (1.20–1.35)	<0.0001	1.14 (1.06–1.22)	0.0003

Recipient gender	Male versus female	1.16 (1.10–1.23)	<0.0001	1.13 (1.06–1.21)	0.0002

Recipient race	AA versus others	1.20 (1.03–1.39)	0.0019	1.09 (0.93–1.28)	0.2994
	Caucasian versus others	1.05 (0.93–1.20)	0.4128	0.95 (0.83–1.09)	0.4842
	Hispanic versus others	1.17 (1.01–1.35)	0.033	1.05 (0.90–1.22)	0.540

Recipient BMI	>30 versus ≤25	1.32 (1.23–1.42)	<0.0001	1.26 (1.16–1.35)	<0.0001
	25–30 versus ≤25	1.17 (1.10–1.26)	<0.0001	1.14 (1.06–1.23)	0.0004

Donor age	≥60 versus <60	1.26 (1.18–1.35)	<0.0001	1.21 (1.12–1.30)	<0.0001
Etiology of liver disease	ALD versus others	1.26 (1.14–1.39)	<0.0001	1.22 (1.10–1.36)	0.0002
	HCV versus others	1.37 (1.28–1.48)	<0.0001	1.34 (1.24–1.46)	<0.0001
	NASH + cryptogenic cirrhosis versus others	1.53 (1.37–1.70)	<0.0001	1.45 (1.29–1.63)	<0.0001

Rejection	Treated rejection versus no rejection	3.31 (3.02–3.62)	<0.0001	3.51 (3.20–3.84)	<0.0001

Donor history of diabetes	Yes versus no	1.18 (1.08–1.30)	0.0002	1.20 (1.10–1.31)	0.0001

DDLT: deceased donor liver transplant; AA: African American; BMI: body mass index; ALD: alcohol liver disease; HCV: hepatitis C virus; NASH: nonalcoholic steatohepatitis; HR: hazard ratio; CI: confidence interval.

## Abbreviations

UNOS/OPTN: Organ Procurement and Transplant Network/United Network For Organ Sharing
NODAT: New onset diabetes mellitus after transplantation
LDLT: Living donor liver transplant
DDLT: Deceased donor liver transplant
LT: Liver transplantation
HCV: Hepatitis C virus
ACR: Acute cellular rejection
BMI: Body mass index
MELD: Model for end-stage liver disease
NASH: Nonalcoholic steatohepatitis
ALD: Alcohol liver disease
ALF: Acute liver failure
HR: Hazard ratio.
